# Morphological and pathogenic investigation of the emerging fungal threat *Emergomyces africanus*

**DOI:** 10.1128/spectrum.00863-24

**Published:** 2024-06-25

**Authors:** Elaine C. Albergoni, Haroldo C. Oliveira, Leandro Honorato, Alessandro F. Valdez, Bianca G. Sena, Rafael F. Castelli, Ana Julia Curioni Rodrigues, Bruna H. Marcon, Anny W. Robert, Leonardo Nimrichter, Marcio L. Rodrigues

**Affiliations:** 1 Instituto Carlos Chagas, Fundação Oswaldo Cruz (Fiocruz), Curitiba, Brazil; 2 Instituto de Microbiologia Paulo de Góes, Universidade Federal do Rio de Janeiro, Rio de Janeiro, Brazil; Institut Pasteur, Paris, France

**Keywords:** *Emergomyces*, ultrastructure, cell surface

## Abstract

*Emergomyces africanus* is a highly fatal fungal pathogen affecting individuals with advanced HIV disease. Molecular patterns and ultrastructural aspects of *E. africanus* are unknown, and pathogenic models have not been investigated in detail. Since the cell wall of fungi is a determinant for interaction with the host and antifungal development, we characterized the ultrastructural aspects of *E. africanus* and the general properties of cell wall components under different conditions of growth *in vitro* and *in vivo*. We also tested the pathogenic potential of *E. africanus* in a *Galleria mellonella* model of infection. Transmission electron microscopy revealed the common intracellular, ultrastructural features of fungi in association with a thick cell wall. Scanning electron microscopy revealed a smooth cell surface, with no apparent decorative structures. Yeast cultures of *E. africanus* showed the distribution of chitin, chitooligomers, and mannoproteins commonly observed in fungi. However, in mixed microenvironments containing yeast and filamenting forms of *E. africanus*, the detection of chitooligomers was increased in comparison with isolated yeast cells, while the detection of these components in filamenting forms was markedly reduced. These observations were suggestive of the ability of *E. africanus* to change its cell wall composition in response to different microenvironments. Although *E. africanus* was unable to kill *G. mellonella*, this infection model allowed us to isolate infected hemocytes for further analysis of mannoproteins, chitin, and chitooligomers. Once again, the detection of *E. africanus* chitooligomers was markedly increased. These results reveal previously unknown ultrastructural features of *E. africanus* and suggest a high plasticity in the cell wall of this lethal pathogen.

**IMPORTANCE:**

The epidemiology of fungal infections is very dynamic, and novel health emergencies are hard to predict. New fungal pathogens have been continuously emerging for the last few decades, and *Emergomyces africanus* is one of these threats to human health. This complex scenario points to the need for generating knowledge about emerging pathogens so that new therapeutic strategies can be designed. In this study, we characterized the general cellular and pathogenic properties of the emerging fungal pathogen *E. africanus*. Our results reveal that *E. africanus* manifests some of the typical properties of fungal cells but also exhibits some unique characteristics that might be helpful for the future development of therapeutic strategies.

## INTRODUCTION

*Emergomyces africanus* is a recently discovered fungal pathogen causing emergomycosis, which is highly fatal in individuals with advanced HIV disease (1). *E. africanus* is a thermally dimorphic fungus, and its emergent medical importance has led to the development of enhanced diagnostic methods and improved fungal identification (2), along with experimental tools applicable to the analysis of its pathogenic potential (3).

Neglected diseases persist due to shortcomings in science, market dynamics, and public health efforts (4). Scientific failures manifest when there is an inadequate understanding of the pathophysiology of infectious agents and the corresponding host response (4). Both *E. africanus*, the pathogen, and emergomycosis, the disease, are linked to scientific failures. *E. africanus* affects highly neglected populations (1), and little is known about its pathogenic determinants. A rapid search on Pubmed.com using *Emergomyces* as a search keyword revealed that, by the end of January 2024, only 49 articles were available in the literature (https://pubmed.ncbi.nlm.nih.gov/?term=emergomyces&sort=date). This slow pace of knowledge generation contrasts with the dimensions of the human health threat posed by *E. africanus* (1, 2). Therefore, the accumulation of biological knowledge of *E. africanus* is essential for the development of antimicrobial tools. In this sense, most of the cellular and ultrastructural properties of *E. africanus* are unknown.

The morphological characteristics of pathogens are intricately tied to their ability to cause disease (5). Changes in cell-surface composition, coupled with alterations in cell size and shape during morphogenesis, represent a connection between adaptation to distinct environments and virulence (5). In this context, the determination of microbial morphological aspects necessitates optimized and reproducible methods, which have historically contributed to the accumulation of comprehensive knowledge. For example, it was only in the 1930s, with the introduction of transmission electron microscopes and the concurrent development of preparation techniques for biological samples, that electron microscopic imaging of ultrathin sections of embedded bacteria emerged as the preferred method for in-depth examination of bacterial cell walls at high resolutions. Through this methodology, the discrimination between the structures of Gram-positive and Gram-negative bacteria based on morphological differences in images became possible for the first time (6). Microbial surface components are frequent mediators of host-pathogen interactions, illustrating the importance of these structures in pathogenic microbes (7).

The morphological determinants of *E. africanus* are only superficially known, and there are no reports about the ultrastructure of this pathogen. Similarly, the surface components of *E. africanus* are unknown, as well as its pathogenic potential in different hosts. In this study, we identified the morphological and ultrastructural properties of *E. africanus* under different *in vitro* and *in vivo* conditions, with a focus on its surface components. Our results contribute to major knowledge gaps related to this highly fatal fungal pathogen and may provide an elemental basis for the future development of assays aimed at generating tools to combat this emerging fungal threat.

## RESULTS

**Fig 1:**
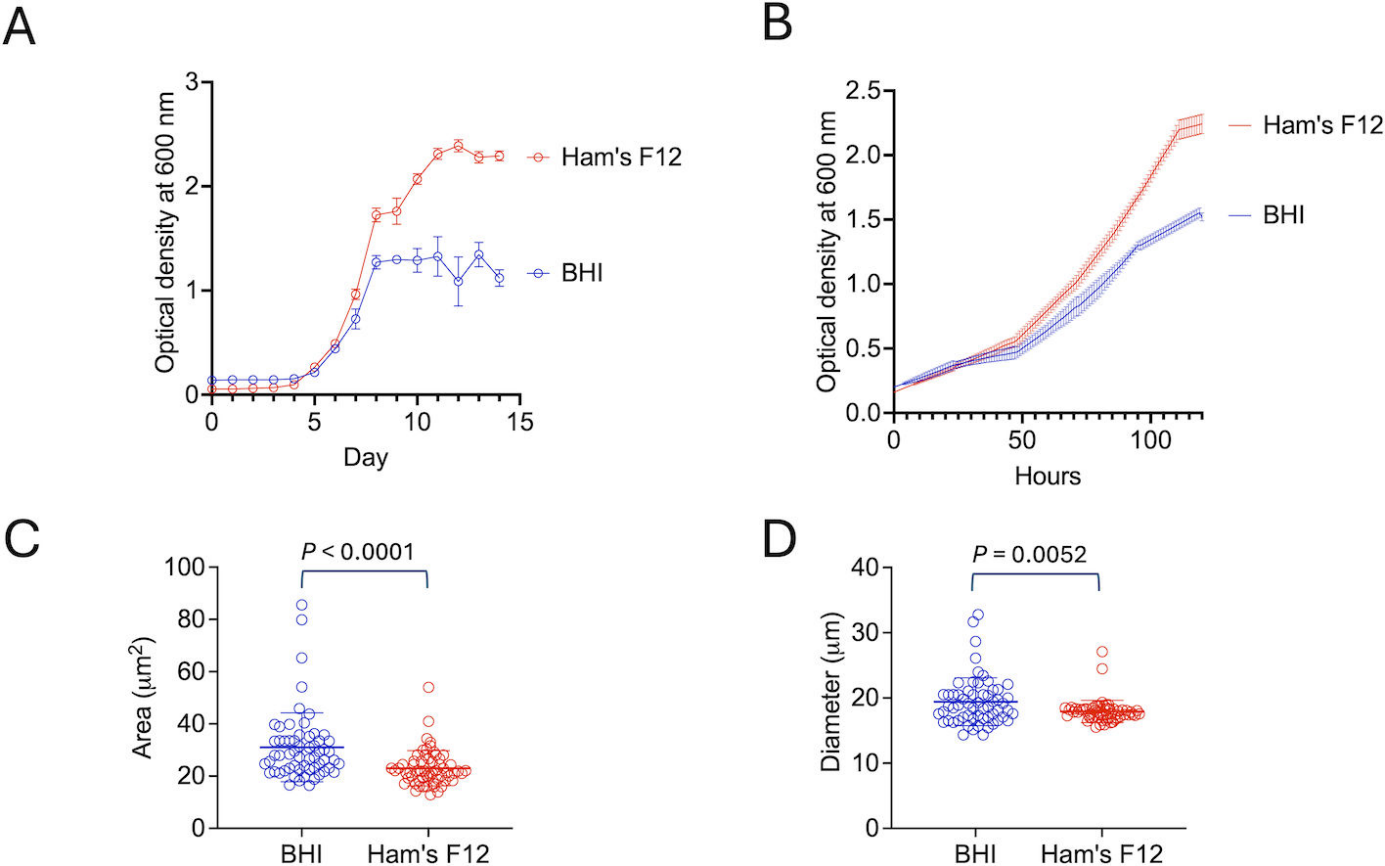
Growth patterns (**A and B**) and cellular dimensions (**C and D**) of *E. africanus* cultivated in Ham’s F12 or BHI. Growth curves in Erlenmeyer flask for 14 days (**A**) and growth in the plate for 4 days (**B**) demonstrated that fungal growth is more efficient in the Ham’s F12 medium. Determination of the cellular area (**C**) and diameter (**D**) indicated that the dimensions of *E. africanus* are larger after growth in BHI in comparison to Ham’s F12 (*P* = 0.0052 for diameter and *P* < 0.0001 for area, as determined by unpaired *t* test).

The ultrastructural aspects of *E. africanus* are unknown. To address this literature gap, we prepared fungal cells for transmission electron microscopy (TEM) after cultivating *E. africanus* in two different liquid media: brain-heart infusion (BHI) and Ham’s F-12 Nutrient Mixture (Thermofisher), supplemented with glucose (1.82 g/L), glutamic acid (1 g/L), 2-(4-(2-hydroxyethyl)-1-piperazinyl)-ethanesulfonic acid (HEPES; 6.5 g/L), and L-Cysteine (8.4 mg/L), both at 37°C to induce the parasitic yeast forms. Our findings unequivocally demonstrated by different approaches that the growth of *E. africanus* was favored in the Ham’s F12 medium (Fig. 1A and B). In addition, the size of the cells was significantly distinct after cultivation in the two different media. We measured both the area and the diameter of fungal cells (Fig. 1C and D), and these parameters revealed that *E. africanus* cells were significantly larger after growth in BHI, in comparison to Ham’s F12 (*P* = 0.0052 for diameter and *P* < 0.0001 for area).

**Fig 2:**
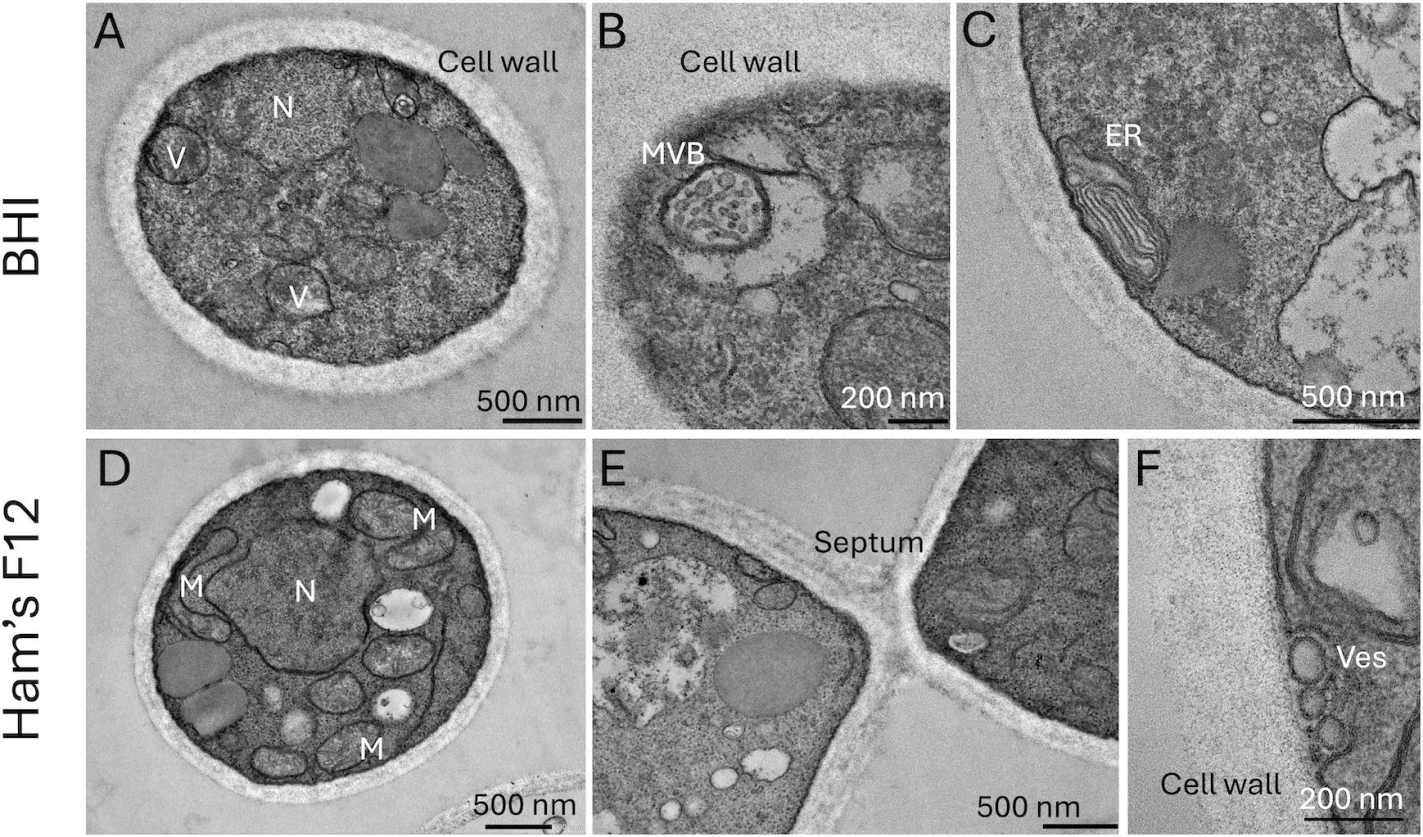
Ultrastructural aspects of *E. africanus* after growth in BHI (**A−C**) or HAM’s F12 (**D−F**) media. Visualization of nuclei (N; **A and D**), a thick cell wall (**A−F**), mitochondria (M; **D**), multivesicular body (MVB)-like structures (**B**), endoplasmic reticulum (ER; **C**), septum (**E**), and vesicles (Ves) in association with the periplasmic space was possible through the use of TEM.

**Fig 3:**
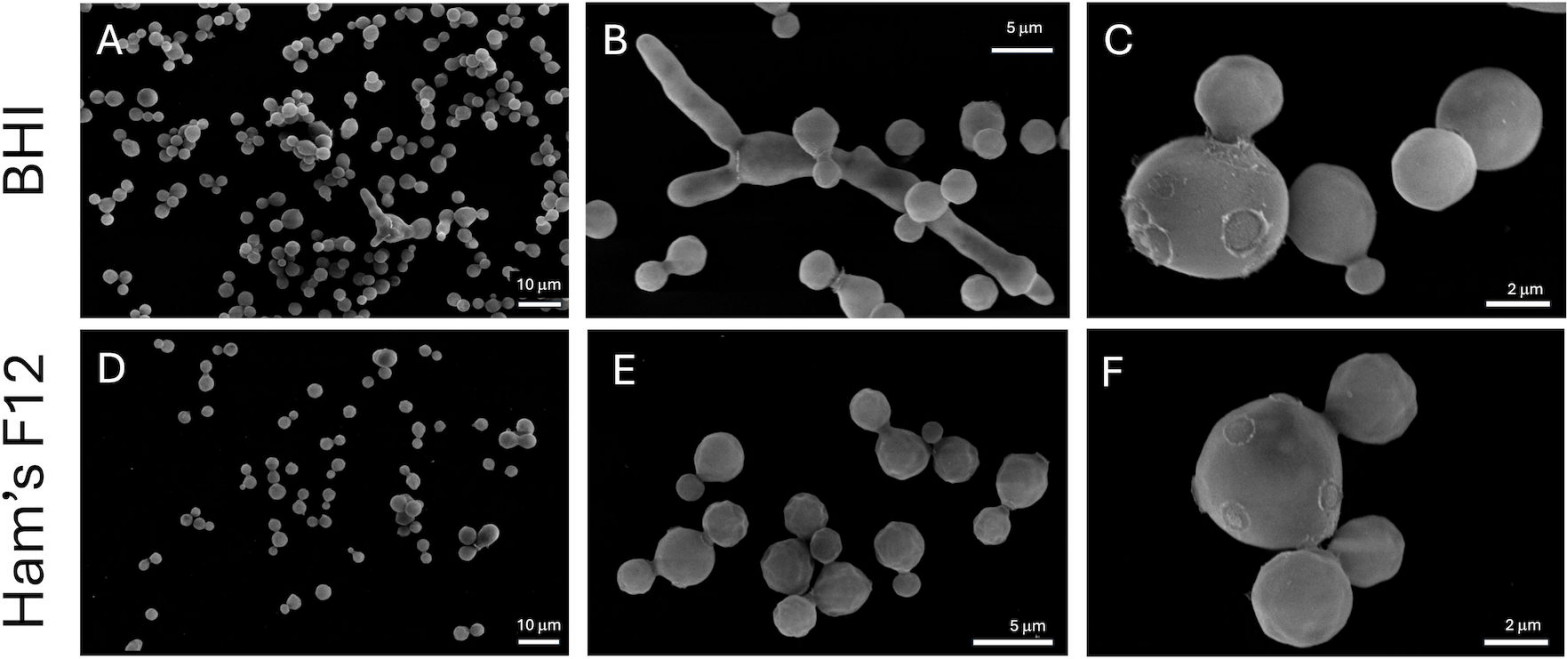
Morphological analysis of the cell surface of *E. africanus* by SEM. The growth of fungal cells in BHI (**A−C**) at 37°C revealed a population mainly composed of yeast cells, with a minor proportion of filamenting fungi. Bud scars in yeast cells were abundantly observed (**C**). When *E. africanus* was cultivated in Ham’s F12 (**D−F**), filamenting forms were not observed. Once again, yeast bud scars were easily evidenced. In all cells, the cell surface manifested as a smooth layer with no apparent decoration.

Despite the differences in size, the cells manifested similar ultrastructural profiles independently of the medium (Fig. 2). In general, *E. africanus* exhibited the usual characteristics of eukaryotic cells. *E. africanus* yeast cells had well-defined intracellular vacuoles and highly electron-dense intracellular membranes, which were often associated with the plasma membrane (Fig. 2A). Several of these vacuoles showed electron-dense structures, and vacuoles resembling multivesicular bodies were observed (Fig. 2B). The plasma membrane was also associated with organelles resembling the rough endoplasmic reticulum (Fig. 2C) and mitochondria (Fig. 2D). Of note, the cell wall of *E. africanus* was consistently found as a thick structure with no apparent surface decoration corresponding to glycans or polysaccharides (Fig. 2A through F), as frequently observed in other yeast pathogens including *Candida*, *Sporothrix*, *Aspergillus*, and *Cryptococcus* (8, 9). The lack of apparent surface decoration was further investigated by scanning electron microscopy (SEM). This analysis revealed that while cultures cultivated in the Ham’s F-12 medium contained only yeast cells, the growth of *E. africanus* in BHI resulted in a mixture of yeast cells and pseudo-hyphae-like forms (Fig. 3). SEM confirmed that the surface of *E. africanus* was smooth, with no apparent cellular projections (Fig. 3A through F). Typical budding yeast cells with evident bud scars were observed in *E. africanus* cells cultivated in BHI or in Ham’s F-12 (Fig. 3C and F).

**Fig 4:**
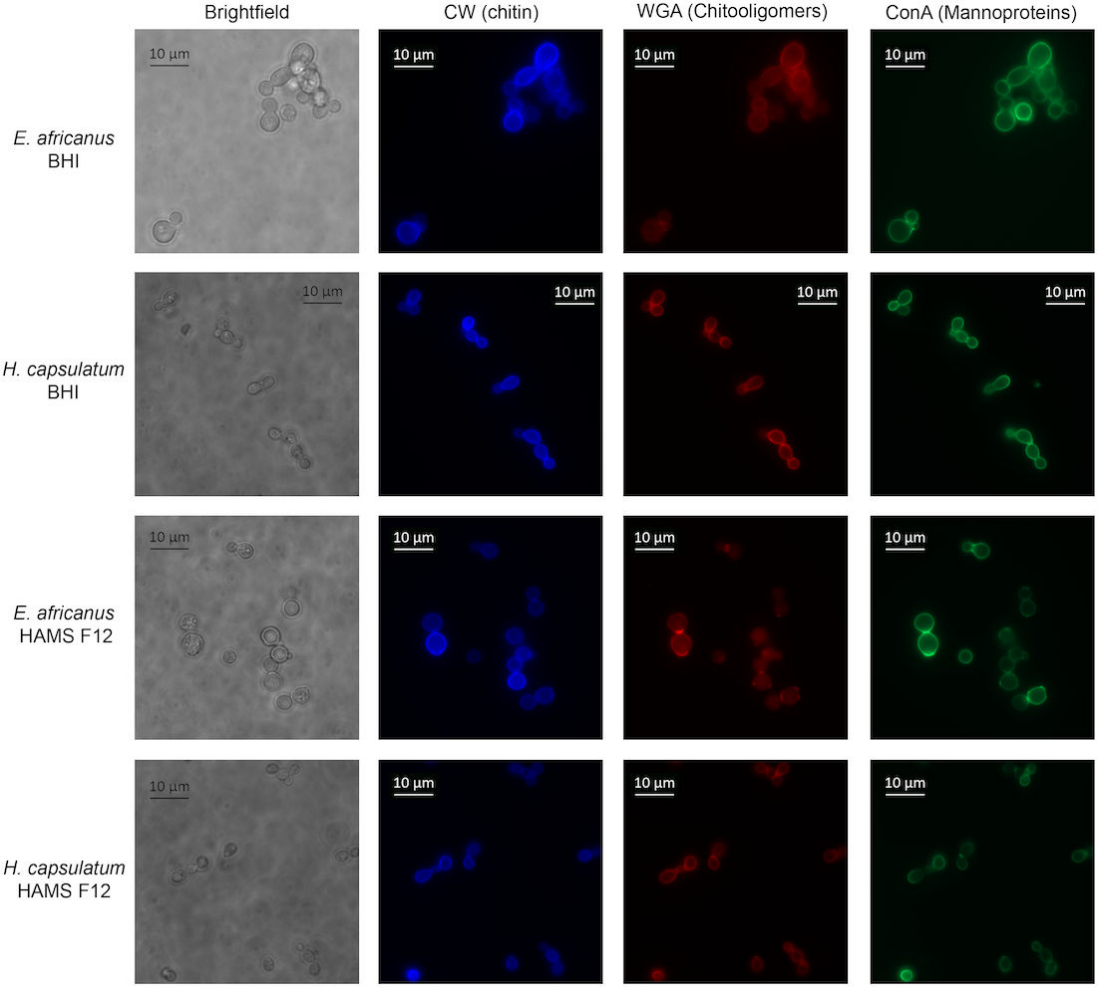
Molecular distribution of cell wall components of *E. africanus* and *H. capsulatum* yeast cells as determined by fluorescence microscopy. Fungal cells cultivated in BHI or Ham’s F12 were stained with calcofluor white for chitin detection (blue fluorescence), WGA for chitooligomer distribution (red fluorescence), or ConA for mannoprotein visualization (green fluorescence). Regardless of the media, fungal cells exhibited the typical annular staining of chitin and mannoproteins at the cell wall. Chitooligomers accumulated at the division sites, as previously demonstrated for other fungi (12).

**Fig 5:**
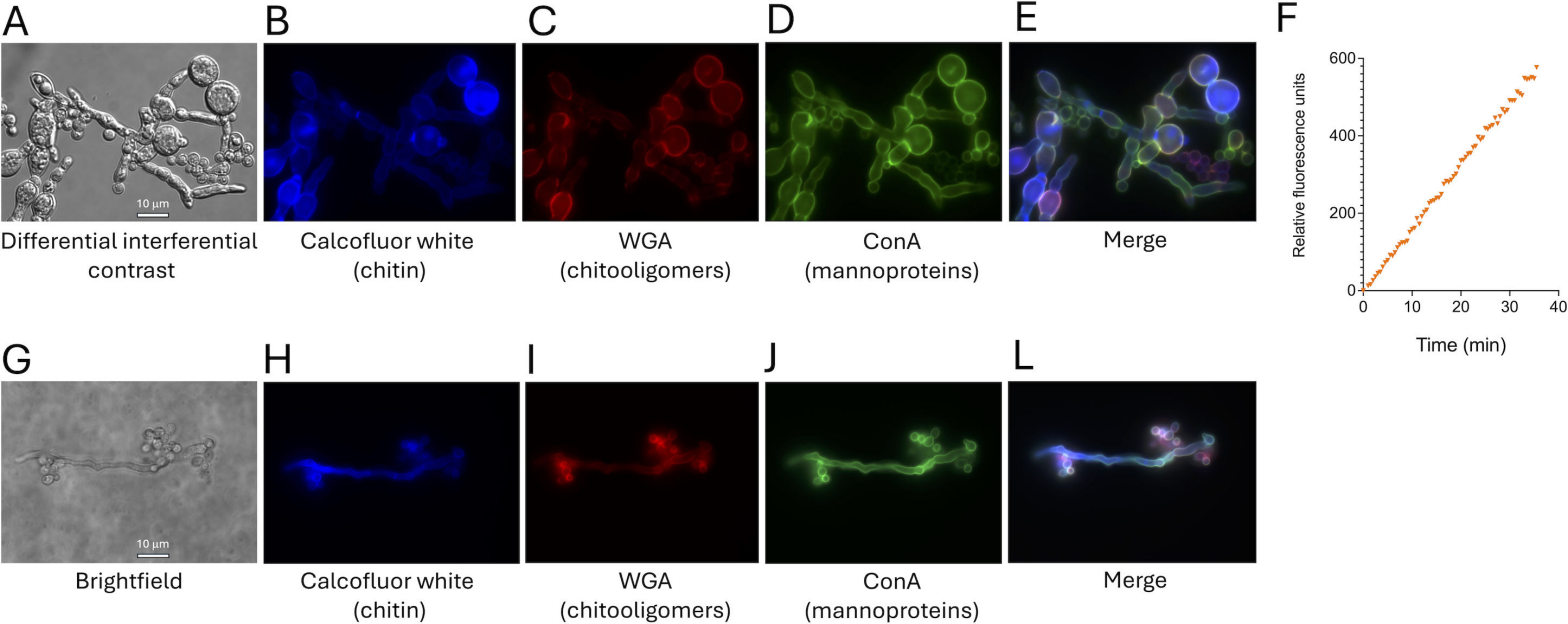
Analysis of morphology, chitin, chitooligomers, and mannoproteins in mixed populations containing yeast and filamenting forms of *E. africanus* (**A−E**) or *H. capsulatum* (**G−L**) grown in BHI, in addition to the determination of chitinase activity in *E. africanus* (**F**). Chitin and chitooligomers were abundantly detected in yeast cells of *E. africanus* (**B, C, and E**) and *H. capsulatum* (**H, I, and L**), although the pattern of WGA binding to this morphological stage (**C and I**) differed from that observed in yeast cells grown in the absence of filamentous forms (see Fig. 4). ConA-binding distribution was apparently not related to morphological transitions (**E and J**). Under these conditions, *E. africanus* cells manifested a time-dependent ability to hydrolyze a chitin-like substrate (**F**). *H. capsulatum* chitinase activity was previously characterized (13) and not explored in this study.

We further used fluorescence microscopy to analyze some of the typical surface components of fungal cells in *E. africanus*, including the presence of mannoproteins, chitin, and chitooligomers (Fig. 4). For this, we used three fluorescent probes, namely fluorescein isothiocyanate-labeled concanavalin A (FITC-ConA, a mannoprotein-binding plant lectin; green fluorescence), calcofluor white (chitin-binding molecule; blue fluorescence), and tetramethylrhodamine isothiocyanate-labeled wheat germ agglutinin (TRITC-WGA, a chitooligomer-binding plant lectin; red fluorescence). The observation of the yeast cells showed that fungi from the BHI medium manifested the typical aspects of fungal cells in respect to their mannoprotein, chitin, and chitooligomer content, consisting of annular staining of the cell wall with calcofluor and ConA, and concentration of WGA staining at the sites of cell division (Fig. 4). Staining of yeast cells after growth of *E. africanus* in Ham’s F-12 produced very similar results. For comparison, we followed the same protocol to stain *Histoplasma capsulatum*, which shares common antigens with *E. africanus* (10), and the results were very similar (Fig. 4). The analysis of *E. africanus* cells grown in BHI confirmed the observation that filamentous forms of the fungus were found under these conditions (Fig. 5). Mixed micropopulations containing filaments and associated yeast cells were also probed for the surface distribution of mannoproteins and chitin-related molecules. Mannoprotein and chitin detection, respectively, followed the pattern observed for yeast cells from the same medium (Fig. 5). Unexpectedly, the detection of chitooligomers by WGA in this condition was very distinct from that observed in yeast cells alone. In filamenting cells, WGA staining was virtually absent, and calcofluor-derived fluorescence was also reduced. In yeast cells associated with the filaments, chitin and chitooligomer detection was abundant and distributed over the entire cell wall (Fig. 5A through E), which contrasted with the punctate pattern observed in isolated yeast cells (Fig. 4). Increased detection of chitooligomers is usually associated with chitinase activity, which degrades chitin to produce chitin-derived oligomers (11). We then asked whether chitinase activity would be detectable in *E. africanus* cells. Indeed, a time-dependent, consistent chitinolytic activity was observed when *E. africanus* was probed for chitinase activity (Fig. 5F). To check whether the decreased chitooligomer detection was specific to *E. africanus*, we once again included *H. capsulatum* in our analysis. Chitooligomer detection and, to a lesser extent, chitin detection were also reduced in filaments but not in yeast cells, with no differences in mannoprotein distribution (Fig. 5G through L). Together, these results were suggestive that the distribution of the surface components of *E. africanus* as well as the fungal morphology was influenced by the microenvironment at which the fungus was present.

**Fig 6:**
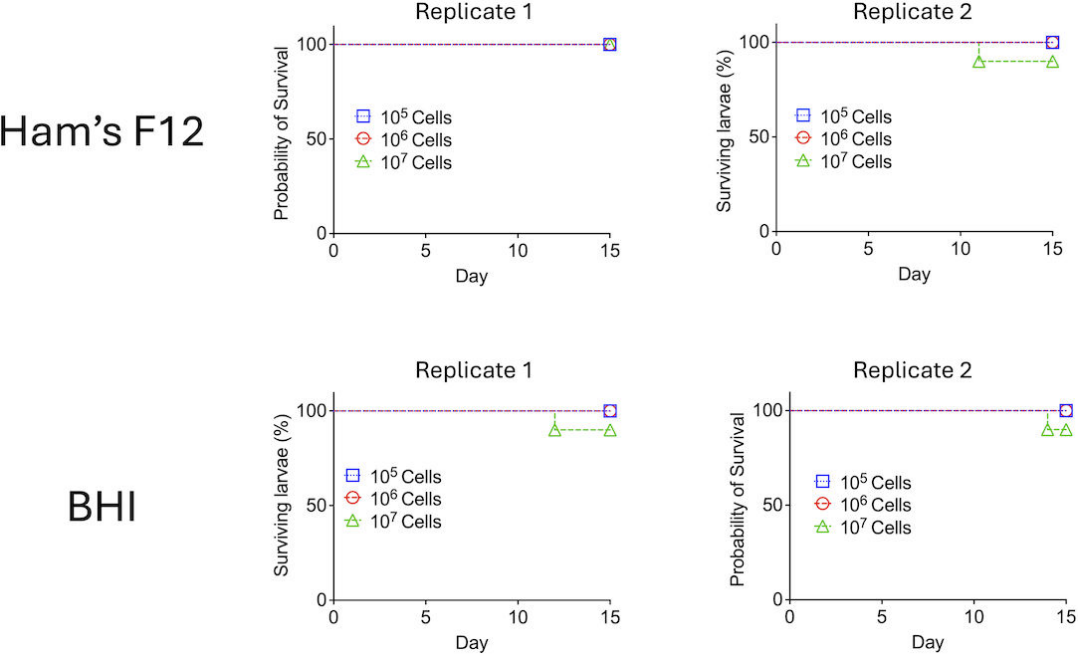
Infection of *G. mellonella* by *E. africanus*. Larvae were infected with varying amounts of *E. africanus* cells grown in Ham’s F12 (upper panels) or BHI (lower panels). Two independent replicates for each experiment are shown. *G. mellonella* was generally resistant to *E. africanus*, as concluded from the negligible mortality independently on the fungal loads used for infection.

**Fig 7:**
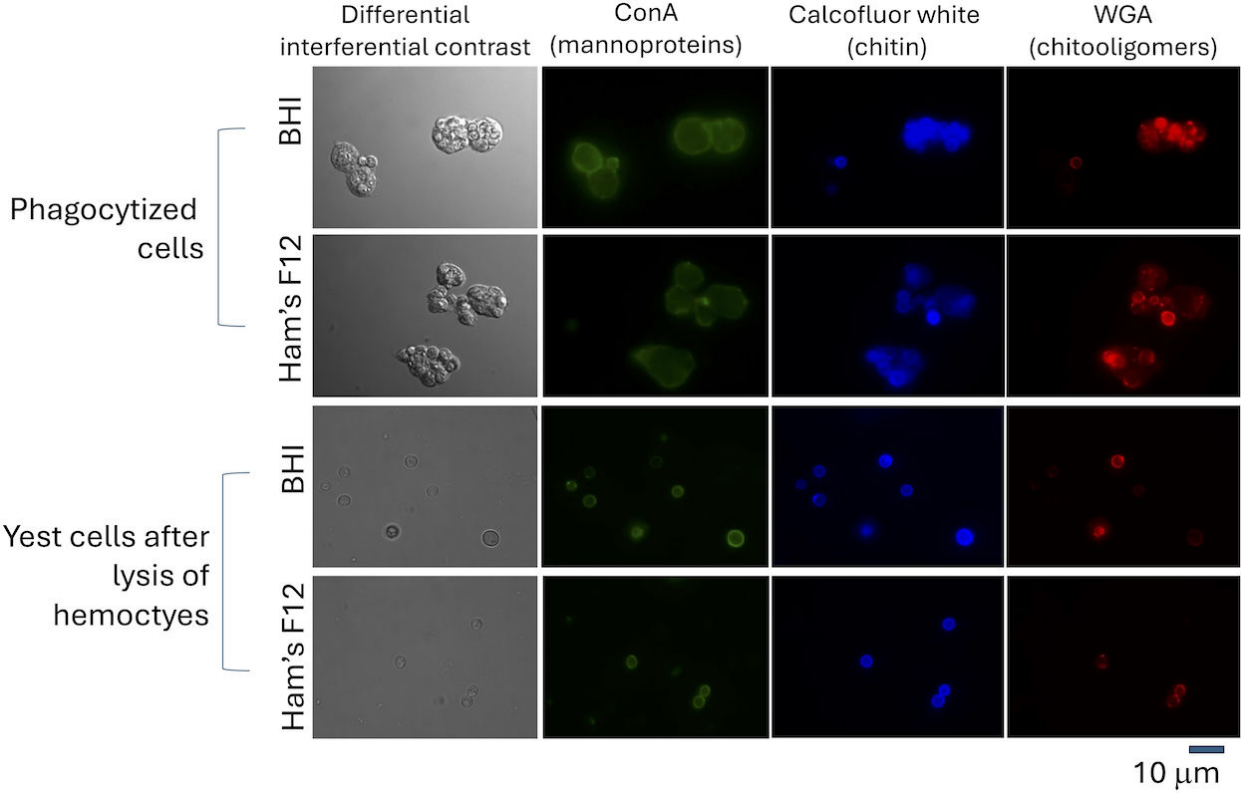
Changes in the cell wall of *E. africanus* in response to the infection of *G. mellonella*. The invertebrate host was infected for 2 h with *E. africanus* (grown in BHI or Ham’s F12), and hemocytes were collected. Infected hemocytes were analyzed by fluorescence microscopy after staining of mannoproteins, chitin, and chitooligomers. Hemocytes infected with *E. africanus* yeast cells were collected (upper panels), but the analysis of changes in the cell wall was hindered by the co-staining of host cell structures. To avoid this interference, hemocytes were lysed, and fungal cells were analyzed alone. Once again, the cell wall response included changes in chitooligomer distribution, which shifted from the punctual pattern observed *in vitro* to an annular pattern of increased detection over the circumference of the cell wall.

The next step in our study was to include a host’s perspective in the morphological analysis. In this sense, we asked whether infection of *Galleria mellonella*, a model host for fungal infections, would change the surface pattern of *E. africanus*. In contrast to what has been described for several other human pathogens (14), *E. africanus* was not able to kill *G. mellonella* (Fig. 6), independent of the fungal dose used to infect the larvae (10^5^, 10^6^, or 10^7^ cells). This observation was suggestive of the existence of controlling mechanisms of the *E. africanus* infection in *G. mellonella*. Therefore, to address our hypothesis that the host could influence the surface molecular pattern of *E. africanus*, we obtained hemocytes, the most effective immune cells of *G. mellonella* (15), 2 h after fungal infection, and searched for the presence of phagocytized fungi. In fact, we observed that hemocytes were abundantly infected with *E. africanus* independently on the medium utilized for fungal growth, but the determination of the fungal molecular patterns was impaired by the fact that hemocytes and fungal cells share common molecules, mainly mannoproteins and, in some cases, WGA-binding structures (Fig. 7A and B). We then lysed the hemocytes to analyze isolated fungal cells. In contrast to fungi cultivated *in vitro*, these cells showed abundant staining for chitooligomers, confirming the notion that the molecular pattern of surface staining in *E. africanus* is dynamic and can change in response to the microenvironment.

## DISCUSSION

The fungal cell wall is one of the most promising targets for antifungal development due to its unique composition in comparison to counterparts in other organisms (7–9, 16). Additionally, the cell wall concentrates several fungal immunogens that are determinant for disease progression or control, in addition to vaccine candidates (7). Therefore, knowledge of the cell wall composition and molecular distribution of its components is essential for antifungal development and understanding the physiopathological steps of fungal diseases. In emergent pathogens, this type of knowledge is commonly not available, as is the case with *E. africanus*. This paradoxical situation, which includes a high medical importance on one side and an evident gap of knowledge on the other, impairs the development of tools applicable to the control of emergent diseases, including diagnostic tests, vaccine prototypes, and novel drugs.

The virulence factors of *E. africanus* are unknown. Indeed, identifying candidates for virulence control demands the characterization of cellular components required for interaction with the host, in addition to regulators of fungal physiology. In this context, our aim in this study was to generate basic information about the biology of the parasitic forms of *E. africanus*. Notably, our results also validate several protocols for the analysis of *E. africanus* that can be used to address different questions involving cellular differentiation, cell biology, analysis of virulence, and fluorescence-based methods, which can be highly strategic for an emerging pathogen associated with largely unanswered questions.

In general, the fungus manifested the cellular properties usually present in other fungal pathogens, including the cell wall architecture, intracellular distribution of organelles, occurrence of surface-associated mannoproteins, wall-associated chitin, and detection of chitooligomers linked to cell division sites. In our study, we used two different media and one infection model to demonstrate that, among the surface components tested, chitooligomers were the ones most prone to modification depending on the environment in which fungal cells were present.

Chitin results from the polymerization of β(1,4)-linked units of N-acetylglucosamine by chitin synthase, which produces chitin microfibrils (17). Chitooligomers, on the other hand, derive from the enzymatic hydrolysis of chitin by endochitinases (chitinases that cleave a chitin molecule at a random point within its chain) and exochitinases (which cleave a chitin molecule at a terminal position) (11). These products of chitin hydrolysis can be detected by WGA, a plant lectin. In general, increased levels of chitooligomers are associated with enhanced chitinase activity (12, 18, 19). Chitin synthesis and degradation must be finely regulated during cellular growth and division, and unbalanced peaks of chitinase activity have been correlated with antifungal effects (20).

The information above is consistent with our results, which show an altered distribution of chitooligomers under different conditions, in association with the detection of chitinase activity. For instance, filamentation requires extensive remodeling of the cell wall (21). In our model, filamentation was associated with the lack of detection of chitin oligomers, suggesting a chitin metabolism oriented more toward synthesis than degradation of the polysaccharide. Filaments originated from yeast forms, which showed, in this environment, increased detection of chitooligomers, suggestive of increased chitinase activity and degradation of chitin to allow cell division. Increased activity of chitinase, however, results in antifungal activity through destabilization of the cell wall (20). In this sense, we speculate that such unregulated activity of chitinase could be related, or partially responsible, to the inability of *E. africanus* to kill *G. mellonella*. In our model, yeast cells that were phagocytized by *G. mellonella* hemocytes were recognized by WGA much more abundantly than cells obtained *in vitro*, suggesting increased degradation of chitin and enhanced production of chitooligomers. Since there are no reports on the existence of chitinase in *G. mellonella*, we speculate that this result derives from the activity of the fungal enzyme. This hypothesis remains to be experimentally confirmed, but these results confirm the plasticity of the *E. africanus* cell wall and its ability to change its molecular distribution in response to different environments. Interestingly, *E. africanus* possibly emerges as a fungal pathogen for which the *G. mellonella* virulence model might not be applicable, in contrast to what has been demonstrated for several pathogenic species of fungi (14). Of note, these conclusions were based on only one isolate. Therefore, we cannot rule out the possibility that this characteristic is specific to the particular isolate investigated in our study.

Several questions related to the biology of *E. africanus* remain open, and this is a clear consequence of a reduced community studying this fungus, which results in reduced scholarly output and several knowledge gaps. In this sense, our study contributes with experimental protocols applicable to the preparation of microscopic samples of *E. africanus*, but also with the general molecular components of its cell surface. Additionally, our results suggest that the cell surface of *E. africanus* is dynamic in its ability to change the distribution of chitin-related components. These molecules have already been demonstrated to be highly immunogenic (22), which could be related to the pathogenic mechanisms used by *E. africanus*.

## MATERIALS AND METHODS

### Strain and growth conditions

*E. africanus* was obtained from a clinical microbiology reference laboratory in the United States. The isolate was identified by Sanger sequencing of the D2 rDNA target, and it was a 100% match to *E. africanus* in the National Center for Biotechnology Information (NCBI) GeneBank database with no other 100% matches to other organisms. The fungus was maintained in BHI broth (Difco) and in Ham’s F-12 Nutrient Mixture (Vitrocell) supplemented with glucose (1.82 g/L), glutamic acid (1 g/L), HEPES (6.5 g/L), and L-Cysteine (8.4 mg/L) referenced from a defined medium called *Histoplasma* macrophage medium (here denominated Ham’s F12) (23) at 37°C, with agitation (200 rpm). For the experiments, *E. africanus* was cultured in both media for 7 days. Growth curves were determined using two different approaches. The cells (10^3^ cells/mL) were inoculated in an Erlenmeyer flask (125 mL capacity) containing 50 mL of BHI or Ham’s F12 and incubated at 37°C with shaking (180 rpm) for 14 days. Fungal growth was measured daily spectrophotometrically on a Spectramax M2 reader (Molecular Device, USA). The experiments were performed as quadruplicates. For the second type of measurement, yeast cells of *E. africanus* were plated onto polystyrene 96-well plates (5 × 10^6^ yeast cells/well) containing BHI or Ham’s F12 (200 µL/well) and cultivated at 37°C. Cellular density was monitored for up to 96 h by measuring the culture absorbance at 600 nm every 1 h using a Hidex Sense Multimode reader (Hidex Sense, Finland). These experiments were carried out as quadruplicates, and similar results were obtained by manual microscopic counting (data not shown). Fungal cells (cultured for 7 days in BHI and Ham’s F12) had their dimensions determined in bright-field microscopy images obtained in an Observer Z1 microscope (Carl Zeiss International, Germany) and analyzed with Fiji ImageJ (version 1.57). Three fields were used, and 60 cells were measured. Statistical analysis of cellular dimensions was performed using an unpaired *t* test with GraphPad Prism software, version 9.0. Fluorescence microscopy, which will be described later in this section, included the analysis of *H. capsulatum*, strain G217b. The fungus was grown for 2 days in an orbital shaker (200 RPM) at 37°C in BHI or Ham’s F-12 medium supplemented with glucose (18.2 g/L), glutamic acid (1 g/L), HEPES (6 g/L), and cysteine (8.4 mg/L), as described previously (24).

### Transmission electron microscopy

*E. africanus* from both BHI (3 × 10^8^ cells/mL) and Ham’s F-12 media (1.55 × 10^8^ cells/mL) was washed three times with phosphate buffered saline (PBS) and fixed in TEM fixative solution adapted for fungal cells (4% paraformaldehyde, 2.5% glutaraldehyde, 0.2M sucrose, and 5 mM calcium chloride in 0.1 M cacodylate solution, pH 7.2) for 5 h at room temperature. The samples were then washed three times with cacodylate buffer, post-fixed with 1% osmium tetroxide in 0.8% potassium ferrocyanide, 5 mM calcium chloride, and 0.1 M cacodylate buffer, and incubated for 1 h at room temperature. Following this, the samples were washed three times with cacodylate buffer, followed by two washes with distilled water. Subsequently, the samples were incubated with 2% uranyl acetate for 1 h and washed three times with distilled water. Dehydration was performed using ethanol in the following concentrations: 30%, 50%, 70%, 90%, and twice with 100% ethanol, with each stage lasting 10 min under gentle agitation. Once dehydration was complete, Spurr low-viscosity resin infiltration was started following these proportions (1 h each): resin 1:2 ethanol; resin 1:1 ethanol; resin 2:1 ethanol. Spurr low-viscosity resin (100%) was then added to the samples, followed by an overnight incubation. Initially, the resin was used without any catalyst. The following day, the samples were infiltrated with 100% Spurr resin with a catalyst for 4 h. Afterward, the cells were incubated in the resin at 60°C for 48 h until the resin was completely polymerized. All infiltration steps were performed on a rotary shaker. Ultrathin sections of 70–80 nm were obtained using a Leica EM UC6 ultramicrotome (Leica, Wetzlar, Germany). The samples were observed using a JEOL 1400 Plus at 100 kV.

### Scanning electron microscopy

*E. africanus* (10^9^ cells/mL) from both BHI and Ham’s F-12 media was washed three times with PBS and fixed overnight with SEM fixative solution [2.5% glutaraldehyde obtained from Sigma Aldrich in 0.1 M cacodylate buffer (pH 7.2)]. Circular coverslips (Knittel) were distributed in a 24-well plate and coated with 0.01% type I poly-L-lysine (Sigma Aldrich) for functionalization for 1 h. The coverslips were then washed three times with dH_2_O and dried at room temperature. Fixed cells were centrifuged at 3,000 × *g* and washed with 0.2 M sucrose, 0.1 M sodium cacodylate buffer, and 2 mM magnesium chloride in dH_2_O three times. The washed samples were adhered to the coverslips for 30 min at room temperature. After adhesion, the sample was submitted to an ethanol dehydration process (30%, 50%, and 70% ethanol solutions for 5 min at each concentration, followed by 90% ethanol and two passes through 100% ethanol, these three stages lasting 10 min each). After dehydration, the cells underwent critical point drying utilizing a Leica EM CPD300 (Leica). Subsequently, they were mounted onto stubs and covered with a delicate layer of gold via a Leica EM ACE200 (Leica) coating apparatus. The examination of the cells was conducted using a scanning electron microscope (JEOL JSM-6010 Plus/LA), at 10 kV, at the microscopy facility situated within the Carlos Chagas Institute, Fiocruz-PR.

### Fluorescence microscopy

*E. africanus* (10^8^ cells) from both BHI and Ham’s F-12 media was washed three times with PBS and fixed overnight in 4% paraformaldehyde in PBS at 4°C. After fixation, the cells were washed three times with PBS. The fungal cell wall was stained with 25 µM calcofluor white (Sigma) for 30 min at 37°C. After this incubation, cells were washed three times with PBS and incubated with 5 µg/mL of succinate WGA-TRITC for 30 min at 37°C. After this incubation, cells were washed three times with PBS and incubated with 25 µg/mL ConA-FITC for 30 min at 37°C. Cells were washed again three times with PBS and suspended in PBS. *H. capsulatum* cells were prepared similarly, except that WGA-TRITC was replaced with WGA-Alexa Fluor 594 at the same concentration. Cells were analyzed under a DMi8 microscope (Leica), and images were recorded with LasAF software (Leica), at the Microscopy Facility at the Carlos Chagas Institute, Fiocruz-PR.

### Chitinase activity

The chitinolytic activity of *E. africanus* cells (5 × 10^6^ cells) was determined using a fluorogenic chitinase substrate, 4-methylumbelliferyl-β-D-N,N',N'-triacetylchitotriose [4MU-(GlcNAc)_3_], which upon reaction releases a fluorescent product, 4-methylbelliferone. This fluorometric assay was based on a protocol previously described (25). For biochemical reactions, 50 µL of samples was added to 25 µL of substrate (50 µM) and 25 µL McIlvaine’s buffer (pH 6.0). The fluorescence produced by the reaction mixture was then measured at 37°C for 30–35 min using a plate reader (SpectraMax M2, Molecular Devices, California, US – 365 nm excitation and 460 nm emission), and the results were expressed in median fluorescence activity (MFI). *Streptomyces griseus* purified chitinase (2.5 × 10^4^ units/mL) was used as a positive control (data not shown). Activity assays were performed in duplicate.

### Morphological aspects of *E. africanus* after interaction with *G. mellonella*

To evaluate changes in the cell surface of *E. africanus* after passage through the host, the invertebrate model of *G. mellonella* infection was used (15). *G. mellonella* used in this study was maintained and cultivated in our laboratory. The larval phase of *G. mellonella* was fed with wax and pollen and kept at 28°C. For the experiments, larvae weighing between 100 and 200 mg were selected and incubated at 37°C overnight, shielded from light and without food. Prior to the experiment, the pro-leg region was cleaned with 70% ethanol. For mortality curves, a Hamilton syringe (Hamilton) was used to inject 10 µL of PBS containing from 10^5^ to 10^7^ cells of *E. africanus* from both BHI and Ham’s F-12 media. As a control, a group of larvae injected with only PBS was used. Each group was composed of ten larvae. Injected larvae were placed in sterile Petri dishes and incubated at 37°C. Survival was monitored daily over a period of 15 days. Larvae were considered dead if they did not respond to physical stimulus. Statistical analysis of the survival curves was performed using the Log-rank Mantel-Cox test with GraphPad Prism software, version 9.0. Alternatively, *G. mellonella* was injected with 10^9^ cells of *E. africanus* in PBS. The different groups were incubated at 37°C for 2 h. After incubation, larvae hemolymph was recovered through a small lesion in the lower part of the larvae with a disposable needle, and the extravasated hemolymph was collected in a 1.5 mL tube, with 1 mL of ice-cold anticoagulant solution (26 mM sodium citrate, 30 mM citric acid, 100 mM glucose, and 140 mM sodium chloride, with pH adjusted to 4.1). To analyze phagocytosis, the content was centrifuged at 400 × *g* for 2 min and washed two times with the anticoagulant solution, followed by fixation with 4% paraformaldehyde. To analyze the phagocytosed cells, after hemolymph extraction, the collected hemolymph in anticoagulant solution was centrifuged at 10,000 rpm for 2 min, and the pellet was suspended in cold sterile dH_2_O and incubated on ice for 20 min to lyse the hemocytes, releasing the phagocytosed *E. africanus*. After incubation, cells were centrifuged for 2 min at 10,000 rpm, and the pellet was fixed with 4% paraformaldehyde. After fixation, cells were washed three times and suspended in PBS. Fixed cells were then processed for fluorescence microscopy with calcofluor white, WGA-TRITC, and ConA-FITC as described in this section.
